# Influenza Virus-Induced Lung Inflammation Was Modulated by Cigarette Smoke Exposure in Mice

**DOI:** 10.1371/journal.pone.0086166

**Published:** 2014-01-21

**Authors:** Yan Han, Man To Ling, Huawei Mao, Jian Zheng, Ming Liu, Kwok Tai Lam, Yuan Liu, Wenwei Tu, Yu-Lung Lau

**Affiliations:** 1 Department of Paediatrics and Adolescent Medicine, Li Ka Shing Faculty of Medicine, University of Hong Kong, Hong Kong Special Administrative Region, People's Republic of China; 2 State Key Laboratory of Respiratory Diseases, Guangzhou Institute of Respiratory Diseases, First Affiliated Hospital, Guangzhou Medical College, Guangzhou, People's Republic of China; University of Tübingen, Germany

## Abstract

Although smokers have increased susceptibility and severity of seasonal influenza virus infection, there is no report about the risk of 2009 pandemic H1N1 (pdmH1N1) or avian H9N2 (H9N2/G1) virus infection in smokers. In our study, we used mouse model to investigate the effect of cigarette smoke on pdmH1N1 or H9N2 virus infection. Mice were exposed to cigarette smoke for 21 days and then infected with pdmH1N1 or H9N2 virus. Control mice were exposed to air in parallel. We found that cigarette smoke exposure alone significantly upregulated the lung inflammation. Such prior cigarette smoke exposure significantly reduced the disease severity of subsequent pdmH1N1 or H9N2 virus infection. For pdmH1N1 infection, cigarette smoke exposed mice had significantly lower mortality than the control mice, possibly due to the significantly decreased production of inflammatory cytokines and chemokines. Similarly, after H9N2 infection, cigarette smoke exposed mice displayed significantly less weight loss, which might be attributed to lower cytokines and chemokines production, less macrophages, neutrophils, CD4^+^ and CD8^+^ T cells infiltration and reduced lung damage compared to the control mice. To further investigate the underlying mechanism, we used nicotine to mimic the effect of cigarette smoke both *in vitro* and *in vivo*. Pre-treating the primary human macrophages with nicotine for 72 h significantly decreased their expression of cytokines and chemokines after pdmH1N1 or H9N2 infection. The mice subcutaneously and continuously treated with nicotine displayed significantly less weight loss and lower inflammatory response than the control mice upon pdmH1N1 or H9N2 infection. Moreover, α7 nicotinic acetylcholine receptor knockout mice had more body weight loss than wild-type mice after cigarette smoke exposure and H9N2 infection. Our study provided the first evidence that the pathogenicity of both pdmH1N1 and H9N2 viruses was alleviated in cigarette smoke exposed mice, which might partially be attributed to the immunosuppressive effect of nicotine.

## Introduction

Influenza A viruses cause regular outbreaks worldwide with significant morbidity and mortality in humans and animals [1], [2]. In 2009, a new swine-origin pandemic H1N1 (pdmH1N1) rapidly spread throughout the world [3]. By 2012, 284,500 deaths were associated with pdmH1N1, majority of which occurred in people younger than 65 years and most of them occurred in Africa and Southeast Asia [4]. H9N2 avian influenza A virus (H9N2/G1) has caused recurrent human infections in Asia since 1998 [5]. Until 2003, 8 cases of H9N2 infection have been reported in China, all with mild influenza-like symptoms and recovered [6], [7]. The new pandemic outbreak of pdmH1N1 and continuous sporadic reports of human infection with H9N2 highlight the ever-existing threat of influenza A virus infection on public health [1], [7], [8].

Cigarette smoke has been considered the main risk factor for morbidity and mortality related to cardiovascular disease, cancer, and chronic obstructive pulmonary disease [9], [10]. Although the adverse effect of cigarette smoke on human health is well documented, there is evidence showing that cigarette smoke might decrease the incidence of some inflammatory and neurodegenerative diseases, including ulcerative colitis, endometriosis, pigeon breeders' disease, sarcoidosis and Parkinson's disease [11]. Mainstream cigarette smoke produces almost 5,000 chemicals, among which, nicotine is mainly responsible for initiation and maintenance of tobacco dependence [12], [13]. Nicotine has immune-suppressive effect by binding to α7 nicotinic acetylcholine receptor (α7 nAChR) and hence activating cholinergic anti-inflammatory pathway [14].

Current epidemiological investigations showed that smokers are at increased risk of influenza virus infection compared with non-smokers [15]–[19], which might be due to the immunosuppressive effect of cigarette smoke. It was reported that such immunologic abnormalities in smokers could be resolved within 6 weeks after cigarette smoke cessation [9]. However, all these studies were on the seasonal influenza (A/H3N2, A/H1N1 and Type B) viruses. Until now, there is no report related to the susceptibility and severity of pdmH1N1 or avian H9N2 influenza virus infection in smokers. As pdmH1N1 and H9N2 can infect mice [20] and mice are widely used to examine the influenza pathology [21], we chose a mouse model to investigate the risk of pdmH1N1 and H9N2 infection in cigarette smoke exposed mice. We hypothesized that the disease severity of pdmH1N1 and H9N2 infection would be exacerbated by cigarette smoke exposure, and we also used nicotine to mimic the effect of cigarette smoke.

## Materials and Methods

### Ethics Statement

Written consent for the use of buffy coat for research purposes was obtained from the donors by the Hong Kong Red Cross Blood Transfusion Services at the time of blood donation. The use of buffy coat for this experiment was approved by the Institutional Review Board of the University of Hong Kong/Hospital Authority Hong Kong West Cluster (IRB reference number: UW 07–390).

All manipulations on animal work in this manuscript were performed in compliance with “Code of Practice for Care and Use of Animals for Experimental Purposes (2004)” and approved by the Committee on the Use of Live Animals in Teaching and Research (CULATR), the University of Hong Kong (reference number: 2631-12 and 2819-12).

### Influenza virus preparation and titration

Influenza virus A/California/04/2009 (pdmH1N1) was grown in embryonated chicken eggs. Influenza virus A/Quail/Hong Kong/G1/97 (H9N2) was propagated in Madin-Darby canine kidney (MDCK) cells with modified Eagle's medium (Invitrogen, Grand Island, NY, USA) containing 2 mg/L N-Tosyl-L-Phenylalanine Chloromethyl Ketone-treated trypsin (Sigma, St. Louis, MO, USA). MDCK cell line was purchased from ATCC (American Type Culture Collection, Manassas, VA, USA) and routinely maintained in our lab. Viruses were purified by ultracentrifugation as described previously [20], [22]. The virus titer was determined by titration in MDCK cells, with daily observation of cytopathogenic effect, and confirmed by hemagglutination assay. The tissue culture infective dose affecting 50% of the cultures (TCID_50_)/ml was calculated by the Reed-Muench formula.

### Smoking mouse model and virus infection

**Figure 1:**
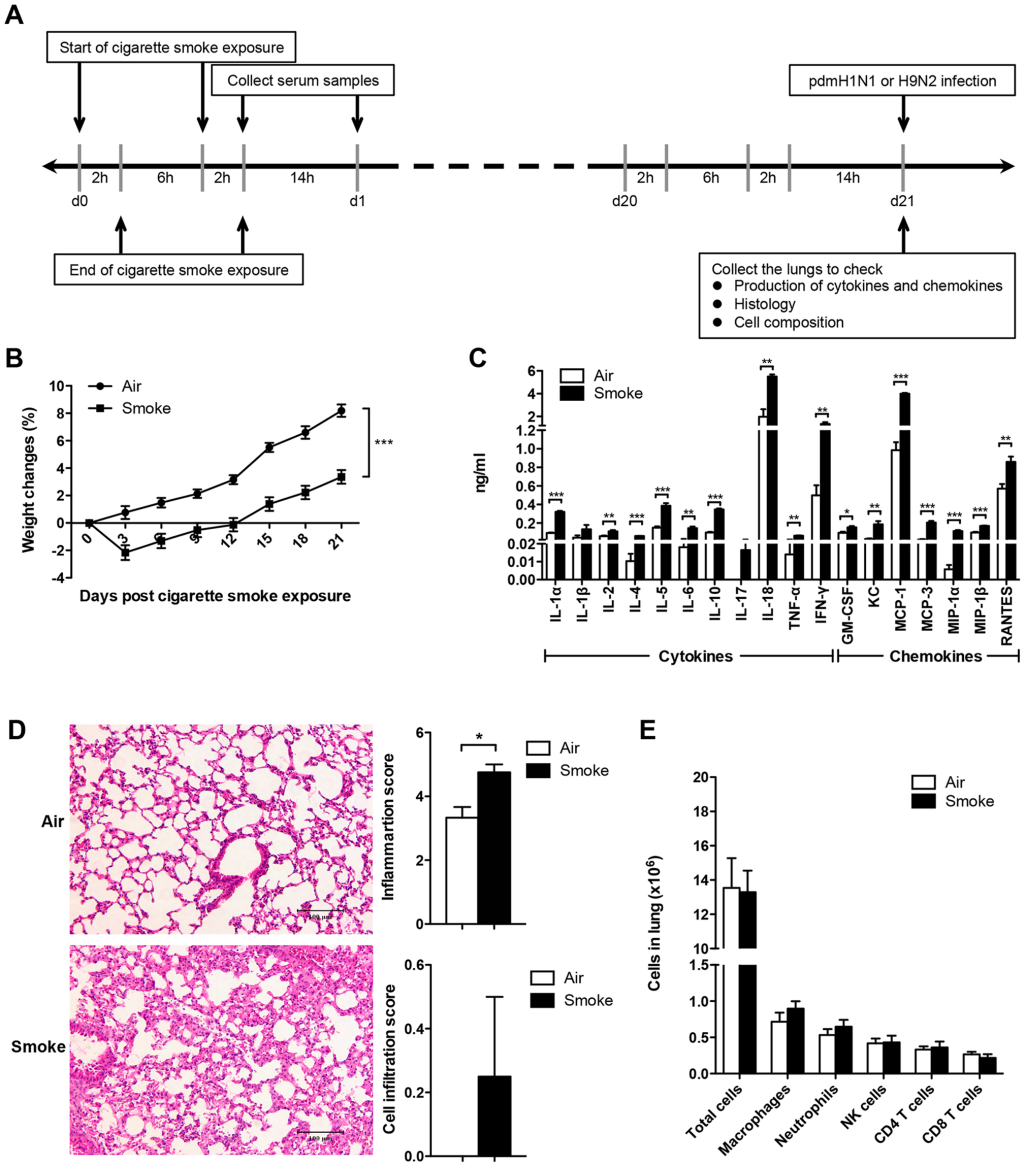
Cigarette smoke exposure alone up-regulated the inflammation of mice. A) Study design for cigarette smoke exposure model. The mice were exposed to 4% (vol/vol, smoke/air) cigarette smoke for 2 hours per episode, 2 episodes per day for 21 days and then infected with pdmH1N1 or H9N2 virus. B) Body weight changes of the mice exposed to room air or cigarette smoke for 21 days. There were 50 mice per group. Data are representative of three independent experiments. C) Production of cytokines and chemokines in lung homogenates on day 21 of air or cigarette smoke exposed mice (fourteen hours after the last cigarette smoke exposure). Data are representative of three independent experiments. D) Histopathological analysis of pulmonary tissues collected on day 21 of air or cigarette smoke exposure (fourteen hours after the last cigarette smoke exposure). Results are representative pictures (200X) of hematoxylin and eosin stained pulmonary tissues. Scale bar: 100 µm. Inflammation score and cell infiltration score were evaluated by a board-certified pathologist. E) Absolute number of lung cells on day 21 of air or cigarette smoke exposure (fourteen hours after the last cigarette smoke exposure). Macrophages (CD11b^+^, F4/80^+^), neutrophils (CD11b^+^, Ly-6G^+^), NK cells (CD3^−^, NK1.1^+^), CD4^+^ T cells (CD3^+^, CD4^+^) and CD8^+^ T cells (CD3^+^, CD8a^+^). There were 4 mice per group (C, D, and E). Results represent mean ± SEM. *p<0.05; **p<0.01; ***p<0.001.

Female C57B6/N mice of 6–8 weeks were obtained from Laboratory Animal Unit, the University of Hong Kong; α7 nAChR knockout mice and wild-type mice were purchased from the Jackson Laboratory. The mice were exposed to 4% (vol/vol, smoke/air) cigarette smoke for 2 hours per episode, 2 episodes per day with commercially available cigarette (Camel; filters, Japan Tobacco INC) for 21 days (Fig. 1A) using the modified ventilated cigarette smoke exposure chambers as described [23], [24]. Control mice were exposed only to fresh air (0%, vol/vol, smoke/air) in another ventilated chamber with the same procedure. Fourteen hours after the last cigarette smoke exposure, the mice were anesthetized and inoculated intranasally with 30 μl of 5×10^3^ TCID_50_ pdmH1N1, or 10^5^ TCID_50_ H9N2 virus. Simultaneously, control mice were treated with PBS in the same way. The well-being of animals was monitored three times daily after cigarette smoke exposure and influenza virus infection. The criteria that we used to monitor the well-being of the mice including appearance, food and water intake, clinical signs (temperature, cardiac and respiratory rates) and behavior (natural or provoked). The mice were humanely euthanized when they showed any one of the following termination criteria: body weight loss ≥30%, body temperature ±2°C, cardiac/respiratory rate ±50%, and sign of severe pneumonia (very weak and pre-comatose). All animal care and experimental protocols were conducted in accordance with the CULATR guidelines of the University of Hong Kong.

These results demonstrated that the peak mortality in the control mice on day 5 post-pdmH1N1 infection might partially be attributed to the hyper-cytokine response. This mortality was significantly reduced by cigarette smoke exposure, which might be due to the significantly decreased production of inflammatory cytokines, as well as reduced pulmonary tissues pathology. The immunosuppressive effect of cigarette smoke exposure was independent of virus replication.

### Quantitation of cotinine

The half-life of nicotine is very short and will be metabolized into different metabolites. The average eradication half-life of nicotine in mouse plasma is approximately 6–7 minutes and cotinine is the major stable metabolite of nicotine [25]. Then serum level of cotinine instead of nicotine was measured using commercially available kit (Neogen, KY, USA) following the manufacturer's instructions. All measurements were performed in duplicate.

### Preparation of mouse lung homogenates

The mouse lungs were harvested at the specific time points and homogenized using a tissue homogenizer (Omni International, Kennesaw, GA, USA) as previously described [20], [26]. The homogenates were centrifuged at 1500 *g* for 10 minutes at 4°C. The supernatants were collected and separated into aliquots. Virus titer was determined immediately and the remaining supernatants were stored at −70°C for cytokines and chemokines detection.

### Cytokines and chemokines determination

Expression levels of interleukin (IL) 1α, IL-1β, IL-2, IL-4, IL-5, IL-6, IL-8, IL-10, IL-17, IL-18, tumor necrosis factor (TNF) α, interferon (IFN) γ, granulocyte-macrophage colony-stimulating factor (GM-CSF), keratinocyte chemoattractant (KC), monocyte chemotactic protein (MCP) 1, MCP-3, macrophage inflammatory proteins (MIP) 1α, MIP-1β, monokine induced by interferon gamma (MIG), regulated and normal T-cell expressed and secreted (RANTES) in the lung homogenates or cell supernatants were quantitatively determined by flow cytometry based immunoassay (eBioscience, San Diego, CA, USA) according to the manufacturer's protocol [20]. The samples were acquired on a LSRII (BD, San Diego, CA, USA) and the amount (ng/ml) was calculated by FlowCytomix^TM^ Pro 3.0 software (eBioscience).

### Pulmonary histopathology

The lung tissues were fixed in 10% formalin, embedded in paraffin, cut and stained with hematoxylin and eosin to analyze inflammation-associated lung damage. Histopathologic inflammation score of lung tissues was evaluated by a board-certified pathologist blinded to experimental design. Lung inflammatory changes were graded using a semi-quantitative scoring system based on the following parameters: peri-bronchiolar and bronchial infiltrates, bronchiolar and bronchial luminal exudates, perivascular infiltrates, parenchymal pneumonia, and edema, as previously described [20], [27]. Each parameter was graded on a scale of 0–4 with 0, absent; 1, slight; 2, mild; 3, moderate; and 4, severe. The total lung inflammation score was expressed as the sum of the scores for each parameter. The degree of cell infiltration was independently scored of 0–3 with 0, no cells; 1, few cells; 2, moderate influx of cells; and 3, extensive influx of cells [20], [26].

To summarize, after H9N2 virus infection, cigarette smoke exposed mice had less weight loss, possibly due to less cytokines production, lower cellular infiltration and less lung damage compared to the control mice.

### Lung cells isolation and flow cytometric analysis

The mouse lungs were cut into small pieces and incubated in Iscove's Modified Dulbecco's Medium (Invitrogen) containing 1mg/ml collagenase D (Roche, Mannheim, Germany) and 20 U/ml DNase I (Roche) at 37°C with shaking for 1–2 hours. After treating with Ammonium-Chloride-Potassium lysing buffer and filtering by a 70 µm nylon mesh, 1×10^6^ cells were stained with a combination of monoclonal antibodies (mAbs) of PE-Cy5-anti-CD11b (M1/70, eBioscience), eFluor 450-anti-F4/80 (BM8, eBioscience), and Alexa Fluor 700-anti-Ly-6G (RB6-8C5, eBioscience); or a combination of mAbs of eFluor 450-anti-CD3 (17A2, eBioscience), FITC-anti-NK1.1 (PK136, BioLegend, San Diego, CA, USA), PE-anti-CD4 (RM4-5, BD), and APC-anti-CD8a (53-6.7, BD); or their relevant isotype-specific antibodies as described previously [26], [28]. Before running samples, counting beads were added into the stained cells (Molecular Probes, Carlsbad, CA, USA). All samples were acquired on LSR II and analyzed by FlowJo software (TreeStar, Ashland, OR, USA).

### Nicotine treatment and virus infection of the cells

Peripheral blood mononuclear cells (PBMC) were isolated from buffy coat of healthy donors (from Hong Kong Red Cross) by Lymphoprep^TM^ (Fresenius Kabi Norge AS) gradient centrifugation. The research protocol was approved by the Institutional Review Board of the University of Hong Kong/Hospital Authority Hong Kong West Cluster. Primary human macrophages were generated from PBMC as described [29]. Briefly, the monocytes were cultured in RPMI 1640 medium (Invitrogen) supplemented with 5% autologous serum and were seeded into the 24-well plates, cultured for 14 days to differentiate into macrophages. The purity of monocytes was examined by flow cytometry (LSRII) with anti-CD14 monoclonal antibody (Invitrogen), and was consistently over 90%. A549 cell line was purchased from ATCC and routinely maintained in our lab. A549 cells were grown in the medium of RPMI 1640 contains 10% fetal bovine serum. A549 cells are cancer cells and are widely used as an *in vitro* model for type II pulmonary epithelial cells [30]. Thus primary human macrophages and A549 cells were used to examine the effect of nicotine on pdmH1N1 or H9N2 virus infection. Primary human macrophages and A549 cells were pre-treated with 10 µM nicotine (Sigma) or PBS for 72 h with the medium changed every 12 h; and then infected with pdmH1N1 or H9N2 (MOI = 2). At 24 h or 48 h post virus infection, the supernatant was collected for cytokines and chemokines measurement.

### Nicotine *in vivo* study

Female C57B6/N mice of 6–8 weeks were anesthetized and implanted with Alzet (Cupertino, CA, USA) micro-osmotic pumps (model 1004) subcutaneously, which provided nicotine at rate of 24 mg/kg/day [31]. Control mice were implanted with pumps filled with sterile water. On day 22 of nicotine exposure, the mice were anesthetized and inoculated intranasally with 30 μl of 5×10^3^ TCID_50_ pdmH1N1, or 10^5^ TCID_50_ H9N2 viruses. After infection, the mice were weighed and monitored every day. The pumps kept deliver nicotine after pdmH1N1 or H9N2 virus infection. All animal care and experimental protocols were conducted in accordance with the CULATR guidelines of the University of Hong Kong.

### Statistical analysis

Data are expressed as mean ± SEM. Statistical analysis was performed by unpaired two-tailed Student's *t test* or one-way analysis of variance (ANOVA) followed by Tukey post hoc test in Prism 5.0 software (GraphPad). The *p* value of the difference for survival was determined by Log-rank (Mantel-Cox) test. The *p* value of the difference for body weight change was examined by multiple regression analysis adjusted for time. A *p* value <0.05 was considered significant.

## Results

### Cigarette smoke exposure alone decreased the body weight gain and elevated lung inflammation

Before investigating the impact of influenza virus infection in cigarette smoke exposed mice, the influence of cigarette smoke exposure alone on mice was determined. Cigarette smoke exposed mice had significantly less body weight gain than the control mice (Fig. 1B). Significantly higher production of IL-1α, IL-2, IL-4, IL-5, IL-6, IL-10, IL-18, TNF-α, IFN-γ, GM-CSF, KC, MCP-1, MCP-3, MIP-1α, MIP-1β and RANTES were shown in cigarette smoke exposed mice than that in control mice (Fig. 1C). Cigarette smoke exposed mice had increased lung damage than control mice, with thickening of intra-alveolar septa and hemorrhages. Moreover, cigarette smoke exposure significantly increased the inflammation score and mildly enhanced the cell infiltration score (Fig. 1D). For the lung cell composition, there were no differences for the number of total cells, macrophages, neutrophils, NK cells, CD4^+^ T cells and CD8^+^ T cells between cigarette smoke exposed mice and control mice (Fig. 1E).

Collectively, cigarette smoke exposure could induce less body weight gain, which might be attributed to up-regulated production of inflammatory cytokines, and increased lung damage compared with the control mice. However, cigarette smoke exposure could not change the lung cell composition.

### Mortality rate of pdmH1N1 infection was decreased by prior cigarette smoke exposure

**Figure 2:**
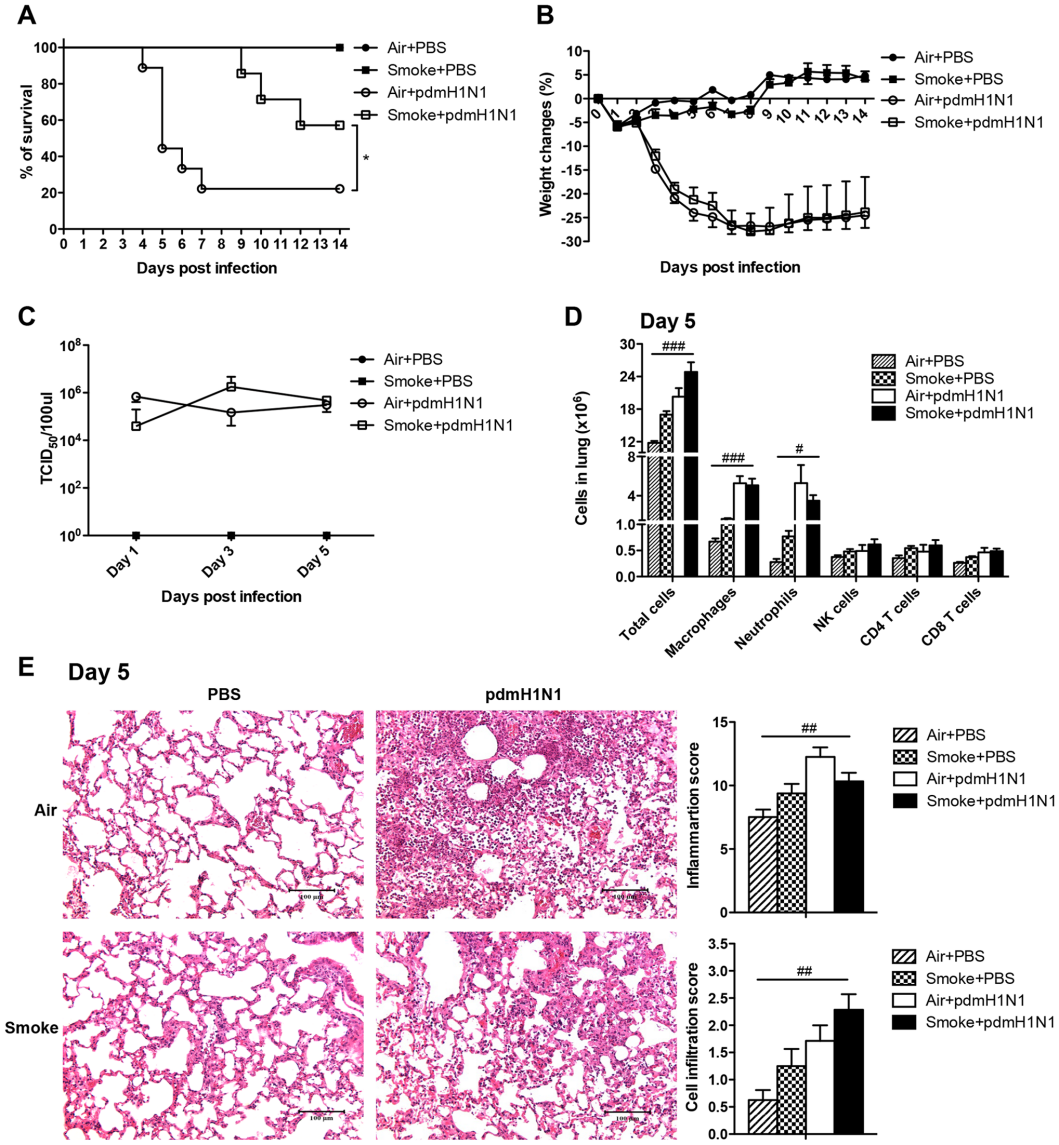
Cigarette smoke exposure decreased the severity of pdmH1N1 infection in mice. The mice were exposed to room air or cigarette smoke for 21 days and then infected with pdmH1N1 virus. A) Survival curve of mice infected with pdmH1N1 virus. There were 6-9 mice per group. Data are representative of three independent experiments. B) Body weight changes of mice infected with pdmH1N1 virus. There were 6–9 mice per group. Data are representative of three independent experiments. C) Lung virus titers of pdmH1N1 infected mice. There were 4–10 mice per group. Data are representative of two independent experiments. D) Absolute number of lung cells on day 5 of pdmH1N1 infection. Macrophages (CD11b^+^, F4/80^+^), neutrophils (CD11b^+^, Ly-6G^+^), NK cells (CD3^−^, NK1.1^+^), CD4^+^ T cells (CD3^+^, CD4^+^) and CD8^+^ T cells (CD3^+^, CD8a^+^). There were 4–5 mice per group. E) Histopathological analysis of pulmonary tissues collected on day 5 of pdmH1N1 infection. Results are representative pictures (200X) of hematoxylin and eosin stained pulmonary tissues. Scale bar: 100 µm. Inflammation score and cell infiltration score were evaluated by a board-certified pathologist. There were 4–8 mice per group. Data are representative of two independent experiments. Results represent mean ± SEM. *p<0.05 was determined by Log-rank (Mantel-Cox) test; ^#^ *p*<0.05, ^##^ *p*<0.01; ^###^ *p*<0.001 were tested by ANOVA of four groups.

**Figure 3:**
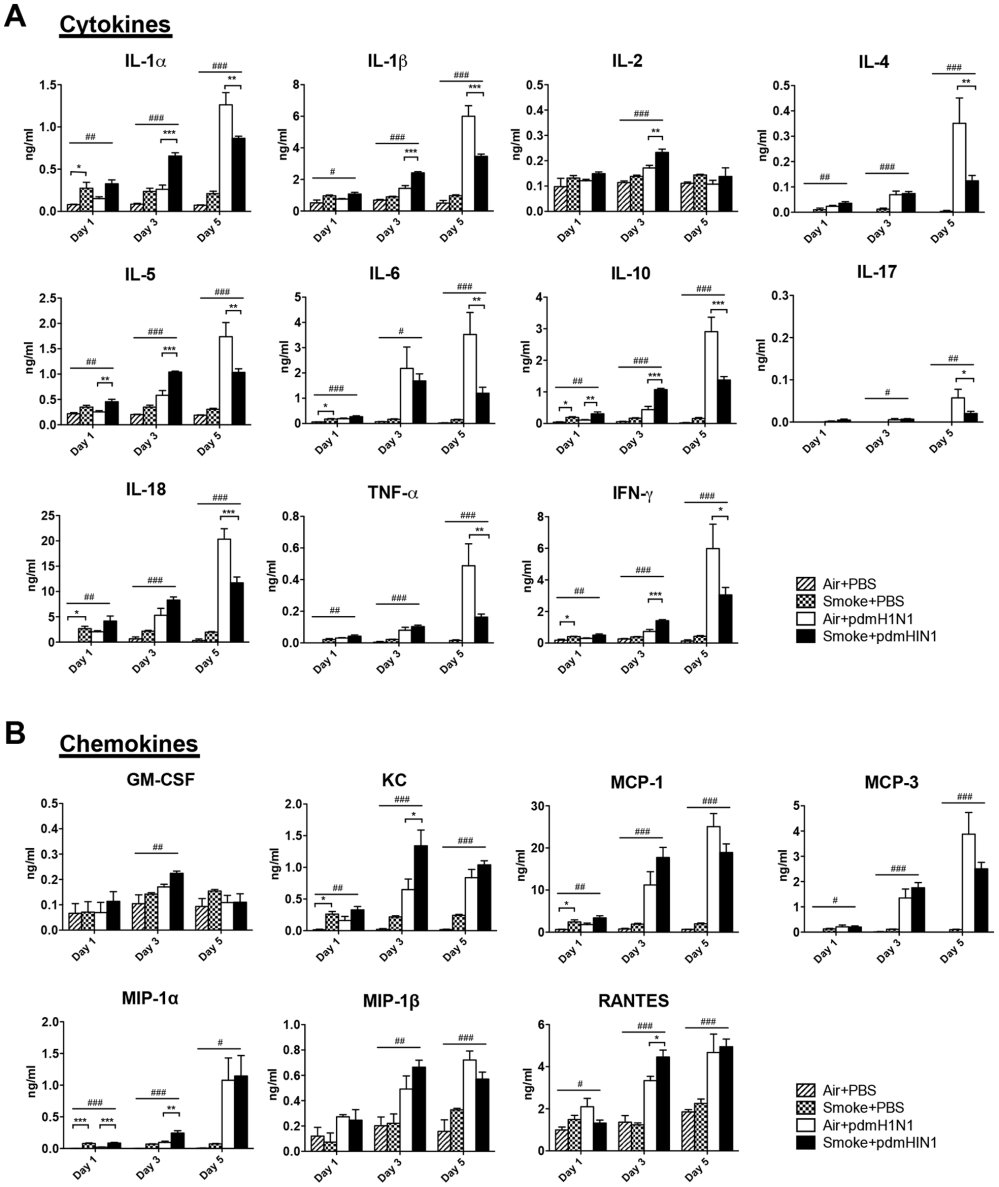
Cigarette smoke exposure suppressed the production of cytokines at later time point of pdmH1N1 infection. The mice were exposed to room air or cigarette smoke for 21 days and then infected with pdmH1N1 virus. The lungs were collected on day 1, 3 and 5 after pdmH1N1 infection. A) The production of cytokines. B) The production of chemokines. Results represent mean ± SEM of 4–10 mice per group. Data are representative of two independent experiments. ^#^ *p*<0.05, ^##^ *p*<0.01; ^###^ *p*<0.001 were tested by ANOVA of four groups; *p<0.05; **p<0.01; ***p<0.001 were determined by Tukey post hoc test.

In order to investigate the role of upregulated inflammation caused by the prior cigarette smoke exposure on the subsequent influenza A virus infection, the mice were infected with pdmH1N1 virus after 21 days of cigarette smoke exposure. Cigarette smoke exposure significantly decreased the mice mortality (Fig. 2A) but had no effect on body weight changes (Fig. 2B) compared to air exposure after pdmH1N1 infection. Because the mice began to die on day 4, early time points of day 1, 3, and 5 after pdmH1N1 infection were selected for the following measurements. There was no difference for lung virus titer at all time points between air and cigarette smoke exposed mice upon pdmH1N1 infection (Fig. 2C). After pdmH1N1 virus infection, cigarette smoke exposed mice had significantly higher production of IL-1α, IL-1β, IL-2, IFN-γ, KC, RANTES on day 3; IL-5, IL-10 and MIP-1α both on day 1 and day 3 in the lungs than the control mice. However on day 5, the phenomenon was reversed, cigarette smoke exposed mice produced significantly lower cytokines than the control mice, including IL-1α, IL-1β, IL-4, IL-5, IL-6, IL-10, IL-17, IL-18, TNF-α and IFN-γ (Fig. 3). For lung cell composition, cigarette smoke exposure could not affect the number of total cells, macrophages, neutrophils, NK cells, CD4^+^ T cells and CD8^+^ T cells in the lung on day 5 after pdmH1N1 infection (Fig. 2D). Less lung damage was observed in cigarette smoke exposed mice on day 5 after pdmH1N1 infection than the control mice; however, the difference was not statistically significant (Fig. 2E).

### Severity of H9N2 infection was reduced by prior cigarette smoke exposure

**Figure 4:**
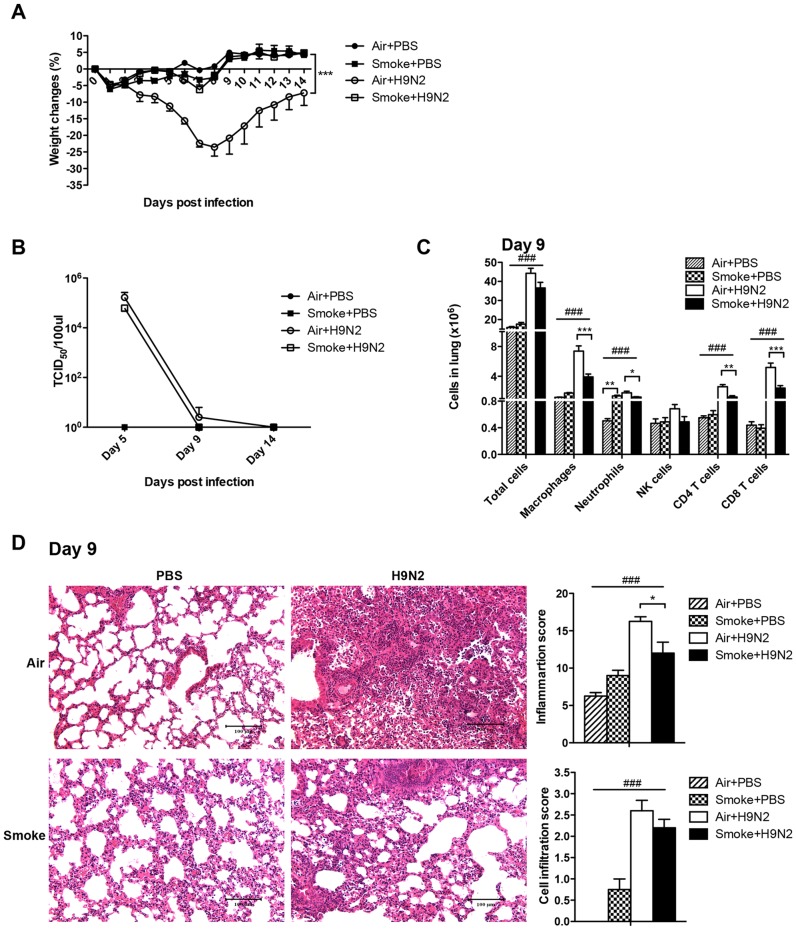
Cigarette smoke exposure reduced the severity of H9N2 infection in mice. The mice were exposed to room air or cigarette smoke for 21 days and then infected with H9N2 virus. A) Body weight changes of mice infected with H9N2 virus. There were 6–7 mice per group. Data are representative of three independent experiments. ***p<0.001 was compared between Air+H9N2 and Smoke+H9N2 and was examined by multiple regression analysis adjusted for time. B) Lung virus titers of H9N2 infected mice. There were 4–7 mice per group. C) Absolute number of lung cells on day 9 of H9N2 infection. Macrophages (CD11b^+^, F4/80^+^), neutrophils (CD11b^+^, Ly-6G^+^), NK cells (CD3^−^, NK1.1^+^), CD4^+^ T cells (CD3^+^, CD4^+^) and CD8^+^ T cells (CD3^+^, CD8a^+^). There were 4–5 mice per group. D) Histopathological analysis of pulmonary tissues collected on day 9 of H9N2 infection. Results are representative pictures (200X) of hematoxylin and eosin stained pulmonary tissues. Scale bar: 100 µm. Inflammation score and cell infiltration score were evaluated by a board-certified pathologist. There were 4 mice per group. Results represent mean ± SEM. ^###^ *p*<0.001 was tested by ANOVA of four groups; *p<0.05; **p<0.01; ***p<0.001 were determined by Tukey post hoc test (C and D).

**Figure 5:**
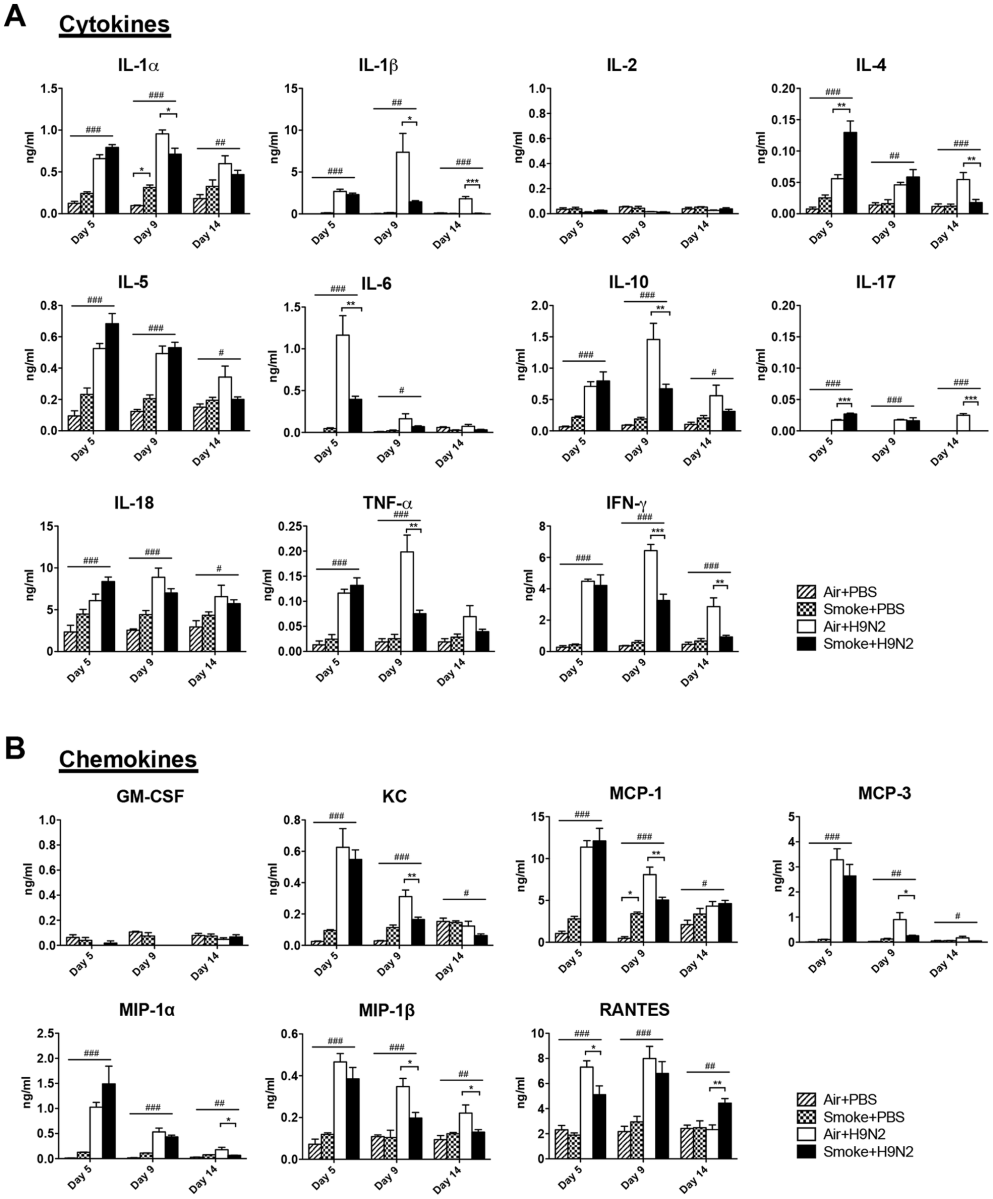
Cigarette smoke exposure suppressed the production of cytokines and chemokines of H9N2 infected mice. The mice were exposed to room air or cigarette smoke for 21 days and then infected with H9N2 virus. The lungs were collected on day 5, 9 and 14 after H9N2 infection. A) The production of cytokines. B) The production of chemokines. Results represent mean ± SEM of 4–7 mice per group. ^#^ *p*<0.05, ^##^ *p*<0.01; ^###^ *p*<0.001 were tested by ANOVA of four groups; *p<0.05; **p<0.01; ***p<0.001 were determined by Tukey post hoc test.

To further investigate whether the immunosuppressive effect of cigarette smoke exposure was independent of influenza subtypes, we tested avian H9N2 virus. Cigarette smoke exposed mice showed significantly lower body weight loss than the control mice (Fig. 4A). There was no death from H9N2 infection; the mice were sacrificed on day 5, 9 and 14 post-infection and the mouse lungs were collected for the following measurements. There was no difference for virus titer at all time points between air and cigarette smoke exposed mice after H9N2 infection (Fig. 4B). For inflammatory response, cigarette smoke exposed mice had significantly lower production of IL-1α, IL-1β, IL-10, TNF-α, IFN-γ, KC, MCP-1, MCP-3 and MIP-1β on day 9, IL-6 and RANTES on day 5 after H9N2 infection compared with the control mice (Fig. 5). Cigarette smoke exposed mice displayed significantly less number of macrophages, neutrophils, CD4^+^ T cells and CD8^+^ T cells than the control mice on day 9 after H9N2 infection (Fig. 4C). Moreover, significantly less lung damage was observed in cigarette smoke exposed mice than in control mice on day 9 after H9N2 infection (Fig. 4D).

### PdmH1N1-induced inflammatory response and disease severity were suppressed by nicotine *in vitro* and *in vivo* respectively

**Figure 6:**
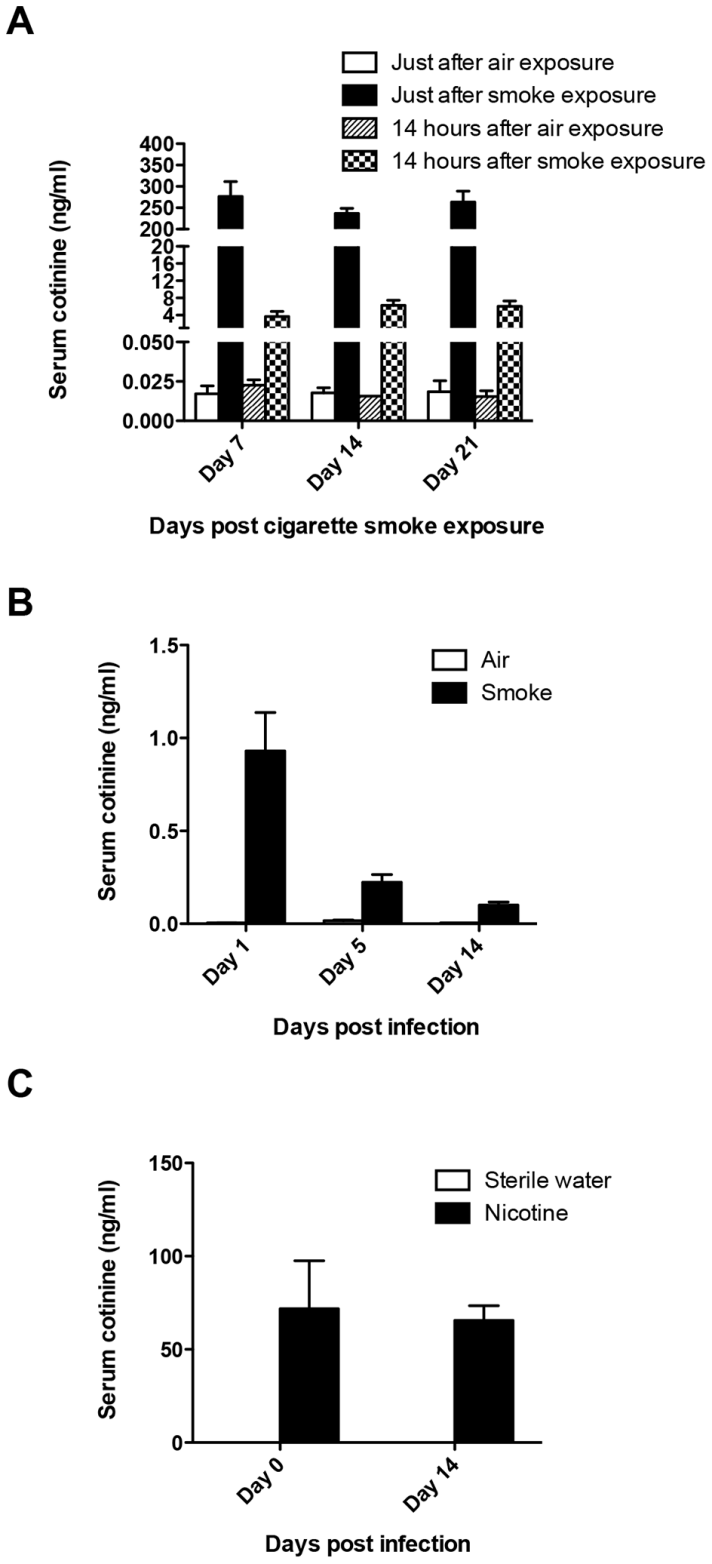
Mouse serum cotinine levels at different time points. A) Serum cotinine levels during 21 days of cigarette smoke exposure. The serum samples were collected on day 7, day 14 and day 21 (immediately or 14 hours after cessation of cigarette smoke exposure). (n = 2 and n = 4 for air and smoke exposure group respectively). B) Serum cotinine levels of air or cigarette smoke exposed mice on day 1, day 5 and day 14 after pdmH1N1/H9N2 virus infection. (n = 2 and n = 4 for air and smoke exposure group respectively). C) Serum cotinine levels of sterile water or nicotine treated mice on day 0 and day 14 after pdmH1N1/H9N2 virus infection. (n = 4 for both sterile water and nicotine treatment groups). Results represent mean ± SEM.

The above data demonstrated that the severity of pdmH1N1 or H9N2 infection was alleviated by cigarette smoke exposure via decreasing the inflammatory response. The underlying mechanisms were further investigated. Nicotine is one of the major components in cigarette smoke but its half-life is short. The serum concentration of cotinine, a major metabolite of nicotine, was about 250 ng/ml, which was comparable to the serum cotinine levels in cigarette smokers [32], immediately after cessation of cigarette smoke exposure, and decreased to around 4 ng/ml at 14 hour after cessation of cigarette smoke exposure (Fig. 6A). Moreover, the mean serum cotinine concentration was 0.93 ng/ml, 0.22 ng/ml and 0.10 ng/ml on day 1, day 5 and day 14 post pdmH1N1 or H9N2 virus infection respectively (Fig. 6B). This data demonstrated that the effect of nicotine might be on-going during the 14 days of pdmH1N1 or H9N2 virus infection. Then we hypothesized that it is nicotine in cigarette smoke that might be responsible for the immunosuppressive effect.

**Figure 7:**
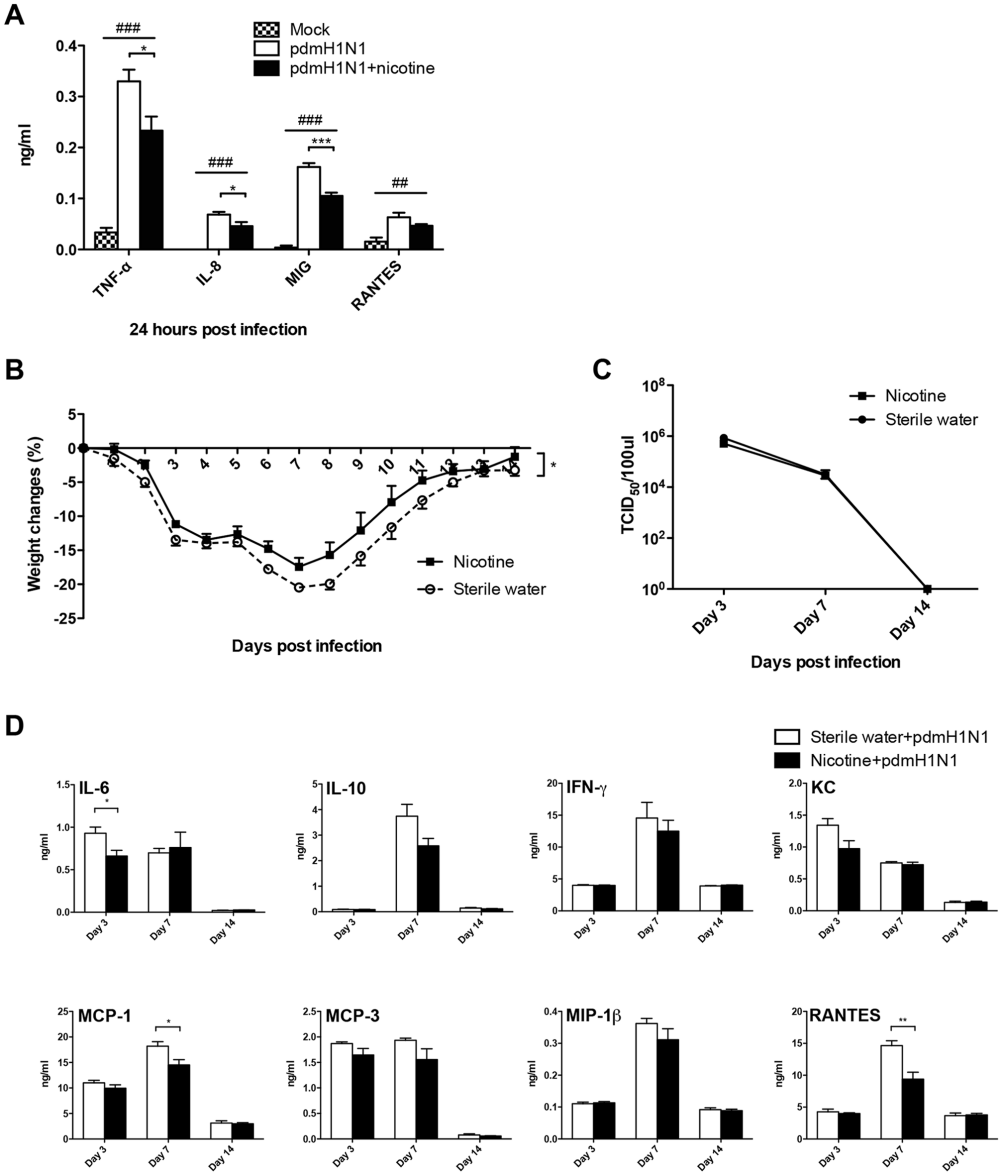
Nicotine suppressed the inflammatory response induced by pdmH1N1 infection *in vitro* and *in vivo*. A) Nicotine inhibited the pdmH1N1-induced production of cytokines and chemokines in primary human macrophages. Primary human macrophages were pre-treated with 10 µM nicotine for 72 h, and then infected with pdmH1N1 virus. Data represent 4–5 independent experiments. ^##^ *p*<0.01 and ^###^ *p*<0.001 were tested by ANOVA of three groups; *p<0.05 and ***p<0.001 were determined by Tukey post hoc test. B) Body weight changes of nicotine treated mice after pdmH1N1 virus infection. The mice were pre-treated with nicotine subcutaneously for 21 days and then infected by pdmH1N1 virus. There were 5 mice per group. Data are representative of two independent experiments. *p<0.05 was examined by multiple regression analysis adjusted for time. C) Lung virus titers of nicotine treated mice at indicated time points after pdmH1N1 infection. There were 4 mice per group. D) Production of cytokines and chemokines in the lung homogenates from nicotine treated mice after pdmH1N1 virus infection. There were 4–5 mice per group. *p<0.05 and **p<0.01 were performed by unpaired two-tailed Student's *t test*. Results represent mean ± SEM.

We firstly used *in vitro* method to test our hypothesis. In primary human macrophages, pdmH1N1-induced production of TNF-α, IL-8 and MIG was significantly inhibited by nicotine (Fig. 7A). In the human lung alveolar epithelial cell line A549, nicotine could decrease the production of MCP-1 at 24 h, IL-8 at 48 h post-pdmH1N1 infection respectively (Fig. S1A). For the *in vivo* study, we chose the dose of 24 mg/kg/day because the serum cotinine concentration was maintained at around 70 ng/ml both on day 0 and day 14 after pdmH1N1 or H9N2 virus infection (Fig. 6C), which was within the range of serum cotinine concentration of the cigarette smoke exposure group (Fig. 6A). Our data demonstrated that there was no death in both sterile water and nicotine delivery groups after pdmH1N1 infection. However, nicotine treated mice had significantly less body weight loss after pdmH1N1 infection compared to control mice (Fig. 7B). There was no difference for lung virus titer at indicated time points between nicotine treated and control mice after pdmH1N1 infection (Fig. 7C). For the inflammatory response, nicotine treated mice had significantly less production of IL-6 on day 3, MCP-1 and RANTES on day 7 after pdmH1N1 infection compared with control mice (Fig. 7D). In summary, the inflammatory response and disease severity induced by pdmH1N1 virus could be suppressed by nicotine.

### H9N2-induced inflammatory response and disease severity were inhibited by nicotine *in vitro* and *in vivo* respectively

**Figure 8:**
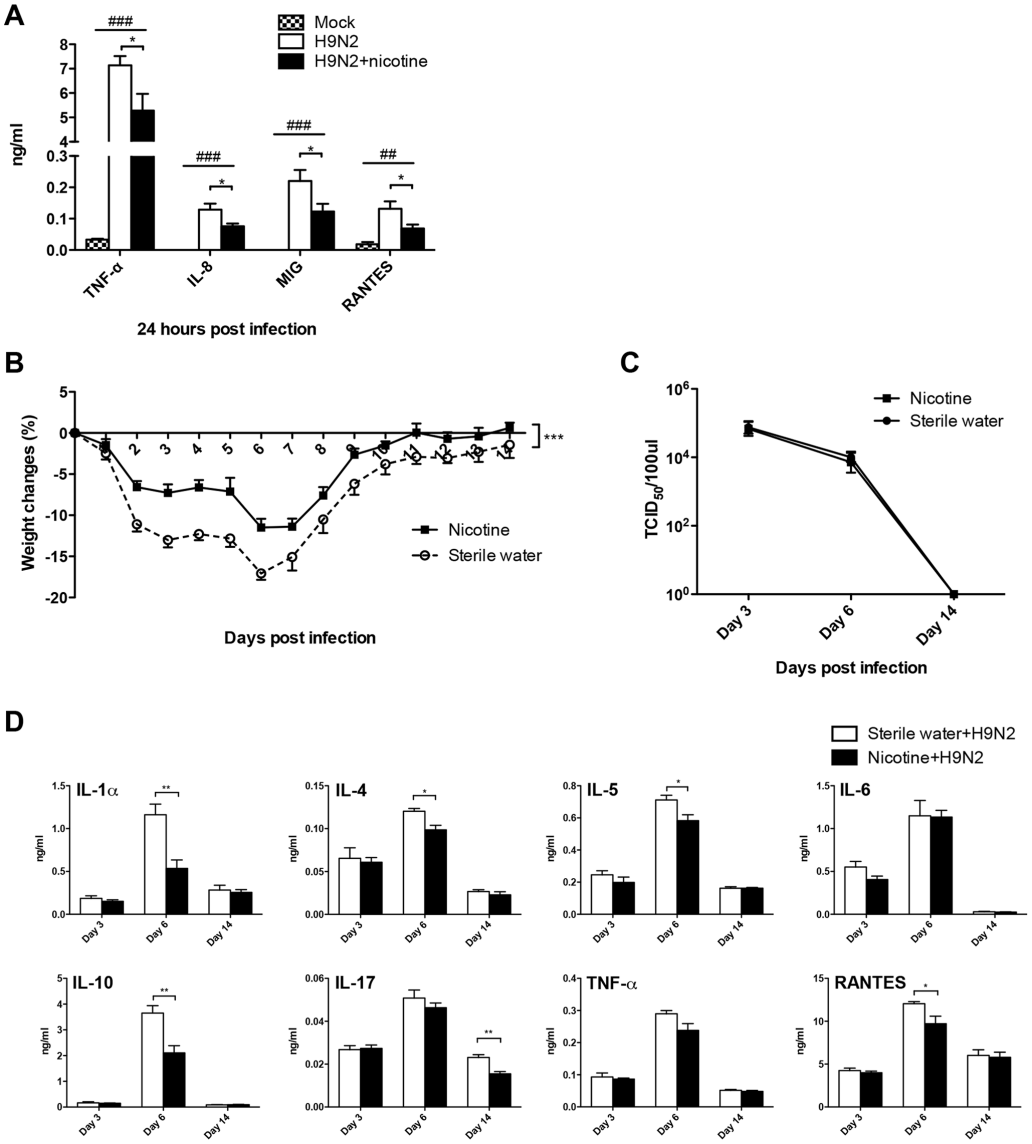
Nicotine inhibited the inflammatory response induced by H9N2 infection *in vitro* and *in vivo*. A) Nicotine suppressed the H9N2-induced production of cytokines and chemokines in primary human macrophages. Primary human macrophages were pre-treated with 10 µM nicotine for 72 h, and then infected with H9N2 virus. Data represent 4–5 independent experiments. ^##^ *p*<0.01 and ^###^ *p*<0.001 were tested by ANOVA of three groups; *p<0.05 was determined by Tukey post hoc test. B) Body weight changes of nicotine treated mice after H9N2 virus infection. The mice were pre-treated with nicotine subcutaneously for 21 days and then infected by H9N2 virus. There were 5 mice per group. Data are representative of two independent experiments. ***p<0.001 was examined by multiple regression analysis adjusted for time. C) Lung virus titers of nicotine treated mice at indicated time points after H9N2 infection. There were 4 mice per group. D) Production of cytokines and chemokines in the lung homogenates from nicotine treated mice after H9N2 virus infection. There were 4–5 mice per group. *p<0.05 and **p<0.01 were performed by unpaired two-tailed Student's *t test*. Results represent mean ± SEM.

We further investigated whether the immunosuppressive effect of nicotine was independent of influenza subtypes and then tested H9N2 virus. H9N2 induced expression of TNF-α, IL-8, MIG and RANTES was significantly suppressed by nicotine in primary human macrophages (Fig. 8A). Nicotine could also suppress the expression of IL-8 at 24 h post-H9N2 infection in A549 cells (Fig. S1B). There was no mortality in both sterile water and nicotine delivery groups after H9N2 infection. However, nicotine treated mice had significantly less body weight loss after H9N2 infection compared to control mice (Fig. 8B). There was no difference for lung virus titer at indicated time points between nicotine treated and control mice after H9N2 infection (Fig. 8C). For the inflammatory response, nicotine could suppress the secretion of IL-1α, IL-4, IL-5, IL-10 and RANTES on day 6 and IL-17 on day 14 significantly after H9N2 infection compared with control mice (Fig. 8D). Taken together, nicotine had immunosuppressive effect on H9N2 infection.

### Cigarette smoke exposure resulted in increased body weight loss in α7 nAChR knockout mice as compared to wild-type mice after H9N2 virus infection

**Figure 9:**
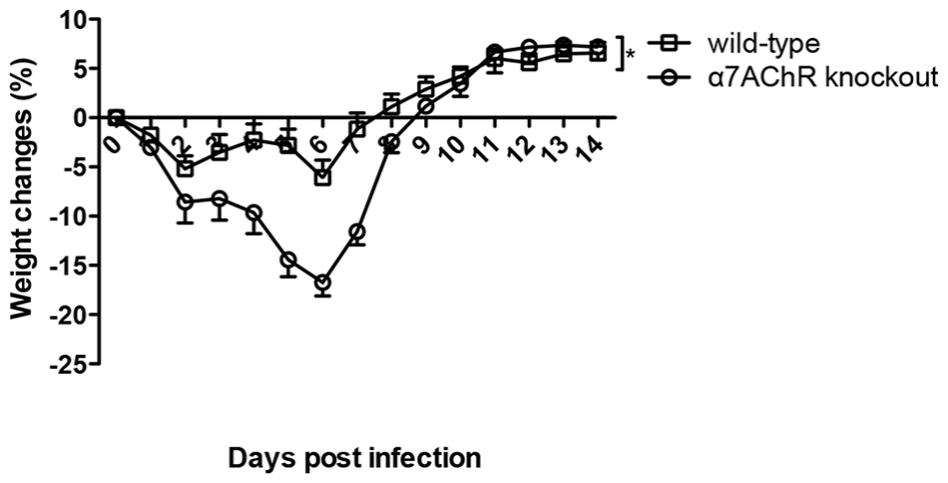
Cigarette smoke exposure resulted in increased body weight loss in α7 nAChR knockout mice as compared to wild-type mice after H9N2 virus infection. Wild-type and α7 nAChR knockout mice were exposed to cigarette smoke for 21 days and then infected with H9N2 virus. The body weight changes were monitored for 14 days. Results represent mean ± SEM. N = 4 and n = 6 for α7 nAChR knockout and wild-type mice respectively. *p<0.05 was determined by multiple regression analysis adjusted for time.

Nicotine exerts its immunosuppressive effect by binding to α7 nAChR and hence activating cholinergic anti-inflammatory pathway [14]. In order to further test the hypothesis that it is nicotine in cigarette smoke that might be responsible for the immunosuppressive effect, we used α7 nAChR knockout mice and wild-type mice to perform the mechanistic study. Wild-type and α7 nAChR knockout mice were exposed to cigarette smoke for 21 days and then infected with H9N2 virus at fourteen hours after the last cigarette smoke exposure. Our data demonstrated that cigarette smoke exposure resulted in significantly increased body weight loss in α7 nAChR knockout mice than in wild-type mice after H9N2 virus infection (Fig. 9).

## Discussion

The present study demonstrated that cigarette smoke exposure alone up-regulated the mouse lung inflammation. The pathogenicity caused by pdmH1N1 or H9N2 virus infection was alleviated by such prior cigarette smoke exposure. The beneficial effect was due to the immunosuppressive effect of cigarette smoke exposure that down-regulated the influenza virus induced hyper-inflammatory response. Indeed, the immunosuppressive effect of cigarette smoke has been previously reported, including macrophages extracted from smokers or smoke exposed mice have reduced inflammation in response to LPS or poly I:C stimulation [33], [34]. Furthermore, this study demonstrated that the immunosuppressive effect of cigarette smoke was partially attributed to nicotine.

Our findings of the beneficial effect of cigarette smoke exposure in pdmH1N1 and H9N2 virus infected mice are contrary to the current epidemiological evidence that showed the mortality and hospitalization rates were increased in smokers who had seasonal influenza infection [15], [16], [19]. This discrepancy could be explained by both viral and host determinants. Both 2009 pdmH1N1 and H9N2 viruses, that crossed species from swine and bird to human respectively, are more virulent than seasonal influenza virus [35], [36]. Therefore, epidemiological data regarding the impact of cigarette smoke on seasonal influenza virus infection might not be applicable to pdmH1N1 and H9N2 virus directly. There is an urgent need to conduct epidemiological investigations to fully understand the impact of smoking on the severity of pdmH1N1 or H9N2 virus infection.

The interaction of cigarette smoke exposure and influenza virus infection in mouse model is complex. Due to the differences in the composition of cigarette smoke, the method and duration of smoke exposure, the dose and subtypes of the influenza A virus, the results of different animal studies might be contradictory. Gualano *et al*., using BALB/C mice exposed to smoke with 9 Winfield Red cigarettes per day for 4 days, and infected with H3N1 virus, found that smoke exposure prior to influenza virus infection led to more lung inflammation, higher viral burden and greater body weight loss [37]. Another study, using C57BL/6 mice exposed to 1R3 cigarettes smoke for 3–5 months and infected with low dose or high dose H1N1-A/FM/1/47, demonstrated that cigarette smoke attenuated the inflammatory response to low dose virus infection, while high dose infection resulted in higher inflammation and significant morbidity [38]. Our study, using C57BL/6 mice, ventilated cigarette smoke exposure system, 21 days of cigarette smoke exposure and pdmH1N1 or H9N2 virus, demonstrated that prior cigarette smoke exposure decreased the severity of influenza virus infection by attenuating the hyper-inflammatory response.

Inflammation is a double-edged sword; adequate inflammation is necessary for the development of immune response, clearance of pathogens and recovery from tissue injuries, while excessive inflammatory response would be life threatening. PdmH1N1 and H9N2 influenza viruses could produce excessive inflammatory cytokines compared with seasonal influenza virus [35], [36], which might contribute to the more severe disease. In support with these observation, here, we also found that, comparing with cigarette smoke exposed mice, control mice had hyper-inflammatory response with higher production of several cytokines, including IL-1α, IL-1β, IL-6, IL-10, TNF-α and IFN-γ on day 5 of pdmH1N1 and day 9 of H9N2 infection, coinciding with the mice having peak mortality and body weight loss respectively. Other studies also demonstrated the deleterious role of dysregulation of cytokines. Enhanced secretion of IL-1α and IL-1β could lead to acute pulmonary inflammatory pathology upon influenza A virus infection in mouse model [39]. Elevated IL-6 levels are associated with disease severity triggered by pdmH1N1 infection in mice [40]. Anti-TNF-α agents could decrease lung inflammation and prolong survival of A/PR/8 infected mice; reduce weight loss and illness severity in mice upon influenza A X31 infection [41]. The expression of IFN-γ is significantly increased in pdmH1N1 infected patients [42]. The deleterious role of hyper-production of IL-10 is supported by IL-10 deficiency mice having enhanced survival upon high-dose influenza virus infection [43]. In our study, cigarette smoke exposure significantly suppressed the production of these inflammatory cytokines after pdmH1N1 and H9N2 infection, leading to the decreased inflammatory response in the lung. The suppressed inflammation can explain the lower mortality after pdmH1N1 infection; less body weight loss, lung injury and immune cells infiltration after H9N2 infection in cigarette smoke exposed mice.

The cell types infiltrating into the lung were mostly neutrophils and macrophages at early time points, with lymphocytes predominant at later time points of infection in our study (data not shown). The hyper-expression of chemokines, such as KC, MCP-1, MCP-3 and MIP-1β in control mice might coordinate the increased recruitment of neutrophils and macrophages on day 9 post-H9N2 infection. Moreover, the increased production of RANTES on day 5, which is chemotactic for T cells, might contribute to higher infiltration of CD4^+^ and CD8^+^ T cells in the control mice at day 9 after H9N2 infection. IL-6, except for its role in resolution of innate immunity, is also an important cytokine to regulate the shift from innate immune response to adaptive immune response and enhance the proliferation of T cells and influenza-specific T memory cells [44], [45]. Therefore, the significantly higher production of IL-6 on day 5 might contribute to the significantly higher recruitment of CD4^+^ and CD8^+^ T cells in the control mice at day 9 after H9N2 infection. The excessive number of macrophages, neutrophils, CD4^+^ and CD8^+^ T cells might lead to deleterious lung immunopathology. By releasing the oxygen radicals and proteolytic enzymes, such as neutrophil elastase and matrix metalloproteinases-8 (MMP-8), MMP-9 and MMP-12, neutrophils and macrophages could cause lung damage [46]. The recruitment of macrophages in the lung could also contribute to the alveolar epithelial cell destruction and apoptosis [47]. More CD4^+^ T cells could induce more inflammatory cytokines, such as IL-6, IL-10 and IFN-γ, leading to more inflammatory response. Furthermore, CD4^+^ T cells could directly lead to severe immunopathology and tissue damage via a cytokine-independent manner in influenza infection [48]. CD8^+^ CTLs could exacerbate influenza viral pathology and induce mortality at high viral dose infection [49]. Our data of significantly increased immune cells number in the lung of control mice upon H9N2 infection corroborated this observation. Cigarette smoke exposure significantly improved this deleterious effect.

In our study, cigarette smoke had no effect on viral burden, which is consistent with other investigations [50]. Some studies however showed increased virus titer in cigarette smoke exposed mice [37] and in smokers [51], [52]. Interestingly, Robbins *et al*. found that cigarette smoke exposure enhanced the viral burden in low dose influenza A virus infection, but had no effect in high dose influenza infection [38]. Taken together, these data indicate that the immunosuppressive effect of cigarette smoke on influenza A virus infection is independent of viral burden.

The immunosuppressive effect of nicotine has been previously reported, including ameliorating inflammatory diseases, such as ulcerative colitis [53], [54] and cutaneous inflammation [55]. Transcutaneous nicotine administration could attenuate the LPS-induced systemic inflammatory response in human subjects [56]. Our *in vitro* and *in vivo* experiments confirmed such immunosuppressive effect of nicotine. Other studies also demonstrated that H1N1-A/PR/8/34-induced morbidity and mortality were decreased by nicotine treatment in mice [57], [58]. However, they found that nicotine promoted influenza infection with increased lung virus titer, which is contrary to our results that nicotine could not affect lung virus replication. Together with our study, these observations suggest that the immunosuppressive effect of nicotine might be independent of virus replication.

For safety consideration, we could not expose the mice to cigarette smoke after pdmH1N1 or H9N2 virus infection. Although the half-life of nicotine is very short, the cotinine could still be detectable on day 14 after pdmH1N1 or H9N2 virus infection. This data demonstrated that the effect of nicotine might be on-going during the 14 days of pdmH1N1 or H9N2 virus infection.

Although we suggested that cigarette smoke exposure decreased the severity of influenza A virus infection, we are not intent to encourage people to smoke. The immunosuppressive effect of cigarette smoke plays a protective role just in our mouse model, when encountering highly virulent influenza virus infection that induces hyper-reaction of inflammatory responses. However, the immunosuppressive effect of cigarette smoke will be deleterious for people suffering from seasonal influenza virus infection [15], [16], [19], which produces appropriate inflammation that is necessary for the generation of immune responses, elimination of pathogens and recovery from tissue injuries [9], [59].

Our study provides the first *in vivo* evidence that cigarette smoke, mediated partially by nicotine, could alleviate the pathogenicity of both pdmH1N1 and H9N2 viruses. Future epidemiological studies of pdmH1N1/H9N2 infection in smokers would be important to clarify the implications of our findings in humans.

## Supporting Information

Figure S1 **Nicotine suppressed the expression of chemokines after pdmH1N1 and H9N2 infection in A549 cells.** A549 cells were pre-treated with 10 µM nicotine for 72 h, and then infected with pdmH1N1 or H9N2 virus. A) Effect of nicotine in pdmH1N1-induced inflammatory response. B) Impact of nicotine on H9N2-induced inflammatory response. Data are mean ± SEM and represent 4 independent experiments. ^#^ *p*<0.05 and ^###^ *p*<0.001 were tested by ANOVA of three groups; **p<0.01 and ***p<0.001 were determined by Tukey post hoc test.
(TIF)
