# Supplementary figures and images for: Influenza Virus-Induced Lung Inflammation Was Modulated by Cigarette Smoke Exposure in Mice
Source: PLoS One. 2014 Jan 21;9(1):e86166. doi: 10.1371/journal.pone.0086166 (PMC3897646; doi:10.1371/journal.pone.0086166)

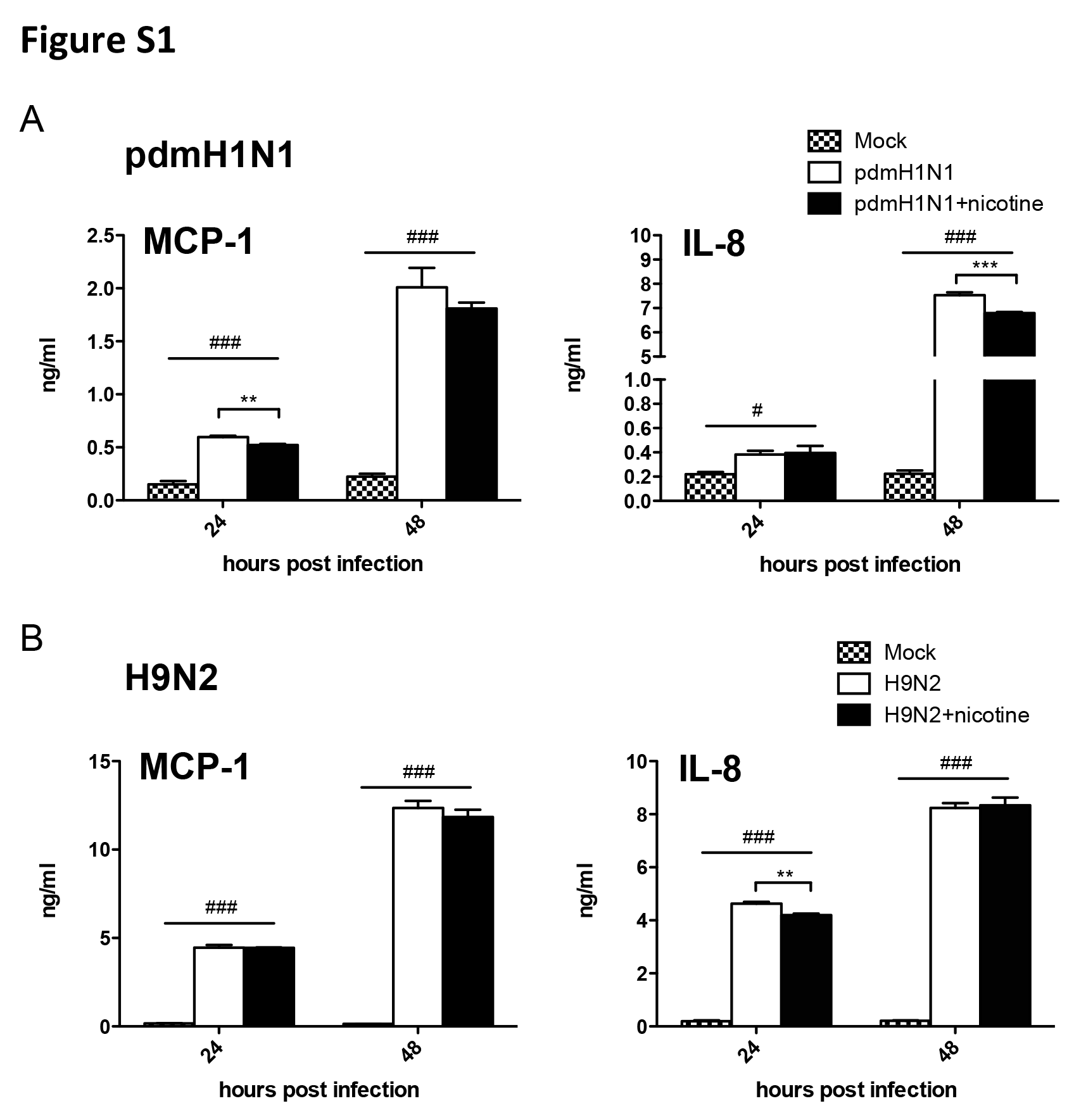

Supplement: Figure S1 — Nicotine suppressed the expression of chemokines after pdmH1N1 and H9N2 infection in A549 cells. A549 cells were pre-treated with 10 µM nicotine for 72 h, and then infected with pdmH1N1 or H9N2 virus. A) Effect of nicotine in pdmH1N1-induced inflammatory response. B) Impact of nicotine on H9N2-induced inflammatory response. Data are mean ± SEM and represent 4 independent experiments. # p<0.05 and ### p<0.001 were tested by ANOVA of three groups; **p<0.01 and ***p<0.001 were determined by Tukey post hoc test. (TIF) [file pone.0086166.s001.tif]
